# A [Mn_8_] Defective Supertetrahedron T3 and Its Dimeric [Mn_16_] Analogue

**DOI:** 10.1021/acs.cgd.4c01100

**Published:** 2024-10-09

**Authors:** Antonis Anastassiades, Dimitris I. Alexandropoulos, Ashlyn Hale, George Christou, Spyros P. Perlepes, Anastasios J. Tasiopoulos

**Affiliations:** † Department of Chemistry, University of Cyprus, Nicosia 1678, Cyprus; ‡ Department of Chemistry, University of Florida, Gainesville, Florida 32611, United States; § Department of Chemistry, University of Patras, 26504 Patras, Greece

## Abstract

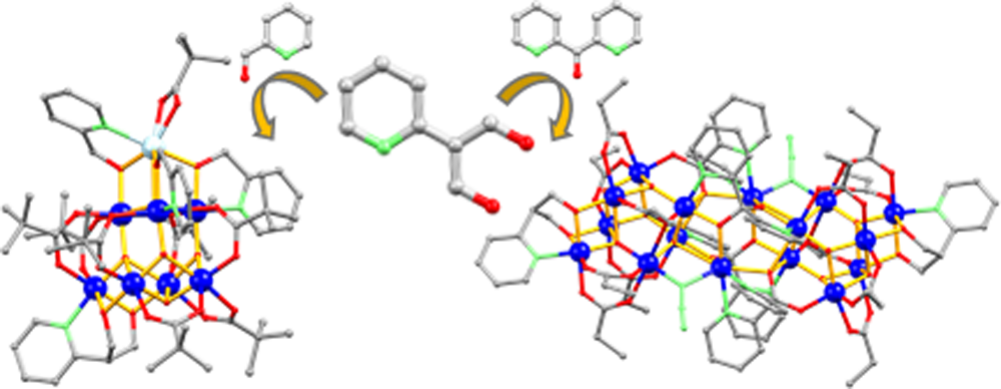

The initial use of 2-(pyridin-2-yl)propane-1,3-diol (pypdH_2_) in Mn cluster chemistry has afforded two new mixed-valence polynuclear Mn clusters, namely, [Mn_8_Ο_5_(pypd)(hmp)_3_(O_2_CCMe_3_)_8_] (**1**) and [Mn_16_Ο_10_(N_3_)_2_(pypd)_2_{(py)_2_CO_2_}_4_(O_2_CEt)_12_] (**2**) (hmp^–^ = deprotonated 2-hydroxymethylpyridine; (py)_2_CO_2_^2–^ = deprotonated gem-diol form of di-2-pyridyl ketone). Compound **1** features a novel [Mn^III^_7_Mn^II^(μ_4_-O)_2_(μ_3_-O)_3_(μ-OR)_5_]^8+^ structural core resembling a supertetrahedron T3, lacking two apexes, while complex **2** has a [Mn^III^_14_Mn^II^_2_(μ_4_-O)_4_(μ_3_-O)_6_(μ-N_3_)_2_(μ_3_-OR)_2_(μ-OR)_8_]^14+^ core consisting of two [Mn_8_] subunits related to **1** and thus is a dimeric analogue of **1**. Direct current (dc) magnetic susceptibility studies revealed the presence of dominant antiferromagentic exchange interactions between the Mn ions in complexes **1** and **2** and small ground state spin values for both compounds. Overall, this work highlights the capability of polyol-like chelates like pypdH_2_ to stabilize high nuclearity 3d metal clusters based on polynuclear building blocks.

## Abstract 2

The initial use of 2-(pyridin-2-yl)propane-1,3-diol ligand in Mn cluster chemistry has afforded two new polynuclear [Mn_8_] and [Mn_16_] clusters. They feature novel, structurally related [Mn^III^_7_Mn^II^(μ_4_-O)_2_(μ_3_-O)_3_(μ-OR)_5_]^8+^ (**1**) and [Mn^III^_14_Mn^II^_2_(μ_4_-O)_4_(μ_3_-O)_6_(μ-N_3_)_2_(μ_3_-OR)_2_(μ-OR)_8_]^14+^ (**2**) cores consisting of one and two (connected) defective supertetrahedra T3, lacking two apexes, respectively. Magnetism studies revealed dominant antiferromagentic exchange interactions and small spin ground state values for both compounds.

## 1 Introduction

The synthesis and characterization of 3d metal clusters with various nuclearities and metal topologies continue to be of great interest due to their potential applications in various research fields,^1−3^ including molecular magnetism.^4−6^ Among the first-row transition metals, manganese (Mn) has yielded many clusters with interesting magnetic properties, including some groundbreaking results. For example, the [Mn_12_Ac]^7,8^ cluster triggered significant interest in this area, while the giant [Mn_84_]^9,10^ and [Mn_70_]^10,11^ torus-like complexes are the highest nuclearity 3d single-molecule magnets (SMMs) reported to date. This is largely due to manganese ions’ ability to exist in multiple oxidation states (II, III, and IV) and their propensity to form high-nuclearity metal-oxo clusters, which often exhibit high ground state spin values and/or SMM behavior.^12−14^

To that end, there is a continuing search for new synthetic methods to produce polynuclear Mn clusters with high nuclearities, novel structural characteristics, and intriguing magnetic properties. The choice of the organic ligand is crucial for developing new synthetic approaches to 3d metal clusters. Therefore, diverse organic chelating/bridging ligands, such as (poly)alcohols^15−18^ or oximates,^19,20^ have been employed either as the only chelate^15−18,20^ or as components of a “ligand blend” approach.^21−23^ These efforts have led to a series of high nuclearity homometallic or heterometallic Mn clusters some of which display impressive structures such as [Mn_29_],^24^ [Mn_31_],^25^ [Mn_44_],^26^ [Mn_21_Ln],^27^ [Mn_26_Ln_6_],^28^ [Mn_36_Ni_4_],^29^ [Mn_32_Co_8_],^30^ and others.^31−34^

In addition, it has been shown that polyols have an exceptional capability to link oligonuclear Mn clusters including triangles, cubanes and others, to form high nuclearity clusters. Apart from oligonuclear (mainly tri- and tetra-nuclear) complexes, well-known higher nuclearity clusters have been recognized as building blocks in polynuclear aggregates.^35−37^ One such example is the [Mn^III^_6_Mn^II^_4_(μ_4_-O)_4_]^18+^ supertetrahedron T3,^18,38−41^ which appears in [Mn_17_]/[Mn_19_],^42,43^ [Mn_25_Na_4_], and [Mn_49_]^44^ clusters displaying two, four and eight edge (or apex in the case of [Mn_19_]) decametallic supertetrahedral subunits, respectively.

**Scheme 1:**
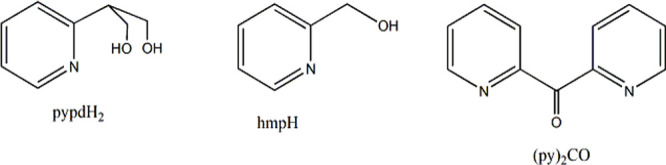
Ligands Discussed in the Text

This significant bridging capability of polyol-type ligands that allowed the preparation of a wealth of high nuclearity clusters has enabled their use in mixed ligand approaches together with chelates with fruitful coordination chemistry. As a result, the combination of polyols with phenolic oximes and various pyridyl alcohols including di-2-pyridylketone ((py)_2_CO), 2-hydroxymethylpyridine (hmpH), pyridine-2,6-dimethanol (pdmH_2_), etc. were investigated. These efforts led to the isolation of several high nuclearity clusters including a [Mn_32_]^22^ double decker wheel, [Mn_23_]/[Mn_24_]^45^capped supertetrahedra T4, and a [Mn_25_]^46^ cluster with a barrel-like cage structure, possessing both ligands employed in the reaction mixture or only one of them. In the latter case, the presence of the second ligand in the reaction system deemed absolutely necessary for the preparation and crystallization of the product. An extension of these efforts involves the study of the polyol ligand 2-(pyridin-2-yl)propane-1,3-diol (pypdH_2_, Scheme 1) not previously employed in Mn coordination chemistry in ligand combination reactions with other pyridyl alcohols, including (py)_2_CO and hmpH.

Herein, the syntheses, crystal structures, and magnetic properties of two new Mn complexes, [Mn_8_Ο_5_(pypd)(hmp)_3_(O_2_CCMe_3_)_8_] (**1**) and [Mn_16_Ο_10_(N_3_)_2_(pypd)_2_{(py)_2_CO_2_}_4_(O_2_CEt)_12_] (**2**), which are the initial results of these investigations, are reported. Complex **1** has a [Mn^III^_7_Mn^II^(μ_4_-O)_2_(μ_3_-O)_3_(μ-OR)_5_]^8+^ defective supertetrahedral T3 structural core, which is unprecedented in metal cluster chemistry, whereas **2** has a [Mn^III^_14_Mn^II^_2_(μ_4_-O)_4_(μ_3_-O)_6_(μ-N_3_)_2_(μ_3_-OR)_2_(μ-OR)_8_]^14+^ core containing two repeating [Mn_8_] subunits, structurally related to **1** in being its “dimeric analogue”. Magnetism studies revealed the existence of antiferromagnetic exchange interactions and small spin ground state values for both complexes.

## 2 Experimental Details

### 2.1 Materials, Physical, and Spectroscopic Measurements

All manipulations were performed under aerobic conditions using materials (reagent grade) and solvents as received. Caution! Although no such behavior was observed during the present work, nitrate and azide compounds are potentially explosive; they should be synthesized and used in small quantities and treated with care. The pyridyl alcohol ligand 2-(pyridin-2-yl)propane-1,3-diol (pypdH_2_) was prepared, purified, and characterized as described elsewhere.^47^ Mn(O_2_CCMe_3_)_2_ and Mn(O_2_CEt)_2_·2H_2_O precursor compounds were synthesized following reported procedures.^48,49^ Elemental analyses (C, H, and N) were performed by the in-house facilities of the University of Cyprus, Chemistry Department. IR spectra were recorded using the ATR technique in the 4000–500 cm^–1^ range employing a Shimadzu Prestige-21 spectrometer. Powder X-ray diffraction data were recorded on a Rigaku Miniflex 6G X-ray diffractometer (Cu Kα radiation, λ = 1.5418 Å). Thermal decomposition studies were performed with a Shimadzu TGA 50 thermogravimetric analyzer. Variable-temperature dc and ac magnetic susceptibility data were collected at the University of Florida using a Quantum Design MPMS-XL SQUID magnetometer equipped with a 7 T magnet and operating in the 1.8–400 K range. Samples were embedded in solid eicosane to prevent torquing. The ac magnetic susceptibility measurements were performed in an oscillating ac field of 3.5 G and a zero dc field down to 1.8 K. The oscillation frequencies were in the 50–1000 Hz range. Pascal’s constants^50^ were used to estimate the diamagnetic corrections, which were subtracted from the experimental susceptibilities to give the molar paramagnetic susceptibility (χ_M_).

### 2.2 Syntheses

[Mn_8_Ο_5_(pypd)(hmp)_3_(O_2_CCMe_3_)_8_] (**1**). Solid Mn(O_2_CCMe_3_)_2_ (0.077 g, 0.30 mmol) was added to a stirred solution of hmpH (15 μL, 0.017 g, 0.15 mmol), pypdH_2_ (0.02 g, 0.15 mmol) and NEt_3_ (42 μL, 0.03 g, 0.30 mmol) in a mixture of MeCN/DMF (2/1 v/v; 15 mL). The resulting brown solution was stirred for 1 h, filtered, and the filtrate left undisturbed at room temperature. After 3 days, dark red/brown crystals of **1**·0.5MeCN·0.5DMF·H_2_O had formed, and they were kept in mother liquor for X-ray analysis, or collected by filtration, washed with MeCN (3 × 5 mL) and dried under vacuum for other solid-state studies. Yield: ∼55%. Anal. calc. (found) for C_68.5_H_106_N_5_O_27.5_Mn_8_ (**1**·0.5MeCN·0.5DMF·H_2_O): C, 43.78 (43.53), H, 5.69 (5.86), N, 3.73 (3.49) %. Selected IR data (KBr, cm^–1^): 3400(m,br), 3150(m,br), 2960(s), 2766(w), 1681(w), 1588(s), 1482(s), 1412(s), 1356(m), 1283(w), 1226(s), 1155(w), 1081(s), 1035(m), 936(w), 891(m), 822(w), 784(m), 719(s), 605(s), 550(w), 516(w), 436(w).

[Mn_16_Ο_10_(N_3_)_2_(pypd)_2_{(py)_2_CO_2_}_4_(O_2_CEt)_12_] (**2**). Solid Mn(O_2_CEt)_2_·2H_2_O (0.072 g, 0.30 mmol) was added to a stirred solution of (py)_2_CO (0.03 g, 0.15 mmol), pypdH_2_ (0.02 g, 0.15 mmol) and NEt_3_ (42 μL, 0.03 g, 0.30 mmol) in a mixture of MeCN/DMF (2/1 v/v; 15 mL). To this solution was then added solid NaN_3_ (0.01 g, 0.15 mmol) under continuous stirring. The resulting brown solution was stirred for 1 h, filtered off, and the filtrate was left undisturbed at room temperature. After 3 days dark red/brown crystals of **2**·MeCN had formed, and they were kept in mother liquor for X-ray analysis, or collected by filtration, washed with MeCN (3 × 5 mL), and dried under vacuum for other solid-state studies. Yield: ∼65%. Anal. calc. (found) for C_98_H_113_N_17_O_46_Mn_16_ (**2**·MeCN): C, 37.44 (37.28), H, 3.62 (3.37), N, 7.57 (7.46) %. Selected IR data (KBr, cm^–1^): 3425(m,br), 3175(w,br), 2976(w), 2940(w), 2367(w), 2081(s), 1667(w), 1587(s,br), 1402(w), 1420(m,br), 1292(s), 1213(w), 1156(m), 1062(m,br), 940(w), 898(w), 816(m), 768(w), 722(m), 606(w,br), 537(w), 457(w).

### 2.3 Single-Crystal X-Ray Crystallography

Data were collected on a Rigaku XtaLAB Synergy-S single crystal X-ray diffractometer equipped with a CCD area detector and a graphite monochromator utilizing Cu Kα radiation (λ = 1.54184 Å). Selected crystals were attached to a glass fiber with paratone-N oil and transferred to a goniostat for data collection. Empirical absorption corrections (multiscan based on symmetry-related measurements) were applied using CrysAlis RED software.^51^ The structures were solved by direct methods using SHELXT,^52^ and refined on *F*^2^ using full-matrix least-squares employing SHELXL-2019/3^52^ and SHELXT.^52^ Software packages used: CrysAlisCCD^53^ for data collection, CrysAlisRED^53^ for cell refinement and data reduction, OLEX2^54^ for geometric calculations, while MERCURY^55^ and Diamond^56^ were used for molecular graphics.

In order to limit the disorder of the terminal ligands or lattice solvent molecules, various restraints (SIMU, RIGU, DELU, DFIX, DANG, ISOR) have been applied in the refinement of the crystal structures. For all compounds, the non-H atoms were treated anisotropically, whereas the H atoms were placed in calculated, ideal positions and refined as riding on their respective C atoms. Unit cell parameters and structure solution and refinement data for complexes **1**·0.5MeCN·0.5DMF·H_2_O and **2**·MeCN are listed in Table S1 of the SI.

## 3 Results and Discussion

### 3.1 Synthetic Comments

Over the past several years, our group has been investigating the use of polyols in manganese carboxylate chemistry. These studies have focused on diols, particularly 1,3-propanediol (pdH_2_) and its derivatives, as a strategy to synthesize high-nuclearity Mn clusters with intriguing magnetic properties. It has been demonstrated that the combination of pdH_2_ with (py)_2_CO leads to the formation of a series of polynuclear complexes, including a family of [Mn_4_M_2_] (M^III^ = Mn, Dy, Gd, Tb) complexes,^57^ based on an uncommon cross-shaped structural core, and [Mn_24_] and [Mn_23_] caped supertetrahedral T4 clusters.^45^ Moreover, the concomitant use of (py)_2_CO with other diols, such as 2-methyl-1,3-propanediol [(m)pdH_2_] or 2-(hydroxymethyl)phenol) (2-hpH_2_) or 1,4-butanediol (1,4-bdH_2_), has resulted in the isolation of several new Mn clusters displaying novel structural features, including some with unprecedented structural cores and interesting magnetic properties.^58^

An extension of these investigations involved the use of a relatively unexplored polyol, 2-(pyridin-2-yl)propane-1,3-diol (pypdH_2_), in Mn carboxylate chemistry. This afforded two new Mn clusters with novel core topologies. To the best of our knowledge there are only a few reports of manganese and copper metal complexes,^59,60^ bearing the methyl substituted pypdH_2_ ligand, LH_2_ = 2-methyl-2-(2-pyridyl)-1,3-propane-diol. In particular, we have investigated the combination of pypdH_2_ with other pyridyl alcohols, including (py)_2_CO and hmpH. We are doing a parentheses here to mention that (py)_2_CO can be considered as mono- or dialcohol^44−46^ because, in the presence of metal ions, normally gives the *gem-*diol form, (py)_2_C(OH)_2_, or the hemiacetal form, (py)_2_C(OR)(OH), the latter in cases where the reaction solvent is an alcohol (ROH; R = Me, Et). Thus, the reaction of Mn(O_2_CCMe_3_)_2_, pypdH_2_, and hmpH in the presence of NEt_3_ in a molar ratio of approximately 1:0.5:0.5:1 in MeCN/DMF produced a brown solution which was filtered off and the filtrate was left undisturbed at room temperature. After approximately 3 days, this solution yielded brown/black crystals of compound **1** in a ∼55% yield. The formation of compound **1** is summarized in eq 1:
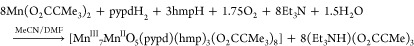
1

The synthesis of **1** involves Mn oxidation, likely by O_2_ under the prevailing basic conditions, and eq 1 has been balanced accordingly. The base Et_3_N facilitates the deprotonation of the pypdH_2_/hmpH ligands and is essential for the synthesis of complex **1**.

Following the isolation of **1**, synthetic efforts were focused on investigating reactions involving other pyridyl alcohols in place of hmpH. However, these reactions did not yield crystalline materials. The addition of azide ions (N_3_^–^), known to be strong ferromagnetic mediators for a wide range of M–N–M angles when bridging in the 1,1-fashion (end-on),^61−63^ and the use of different Mn carboxylate salts as starting materials, led to the isolation of complex **2**. Thus, the reaction of Mn(O_2_CEt)_2_·2H_2_O, pypdH_2_, (py)_2_CO, NaN_3_ and NEt_3_ in a molar ratio of ∼1:0.5:0.5:0.5:1 in MeCN/DMF resulted in a brown solution that was left undisturbed to afford, after 3 days, brown/black crystals of **2** in ∼65% yield. The formation of complex **2** is summarized in eq 2:
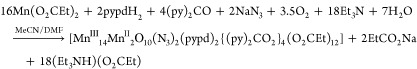
2

The reported compounds were characterized by single-crystal X-ray crystallography, powder diffraction, elemental analyses (C, H, N), thermogravimetric analysis and IR spectroscopy. Details on the IR, pXRD and TGA studies for complexes **1** and **2** are available in ESI (Figures S3–S7 and Table S4).

The IR spectra of the complexes (Figures S5 and S6) are complicated and-no doubt-some bands correspond to more than one vibrational mode. In the spectrum of **1**·0.5MeCN·0.5DMF·H_2_O, the band at 1681 cm^–1^ is attributed to the ν(C=O) mode of the lattice DMF, reflecting its presence in the complex. The strong band at 2081 cm^–1^ in the spectrum of **2**·MeCN is due to the ν_as_(NNN) vibration of the azido group; its wavenumber suggests the end-on bridging character of this group.^64^ The bands at ca. 1590 and 1440 cm^–1^ are tentatively assigned to the ν_as_(CO_2_) and ν_s_(CO_2_) vibrations of the pivalato groups. Their small separation is indicative of their bidentate bridging and chelating nature. The strong bands at ∼790 and ∼610 cm^–1^ in both spectra have been associated with stretching vibrations of the {Mn-(μ_*x*_-O)-Mn} moieties (*x* = 3, 4).^65,66^

### 3.2 Description of Structures

**Figure 1:**
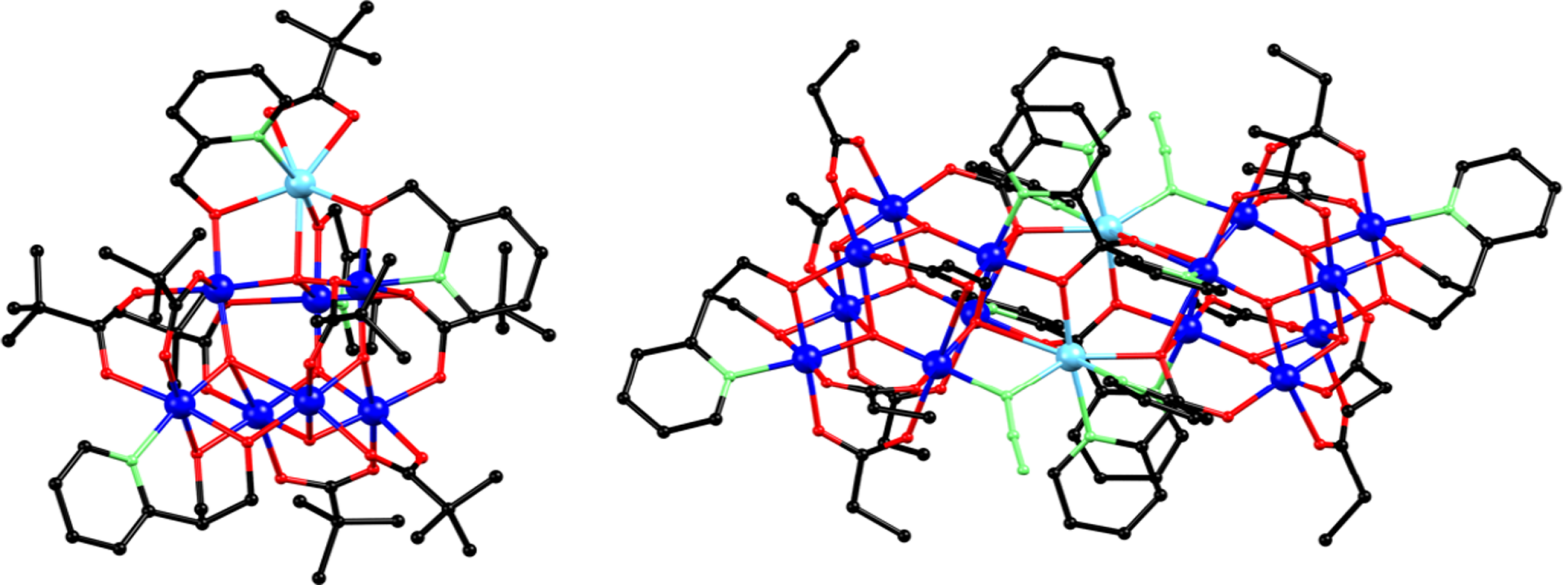
Ball and stick representations of the molecular structure of **1** (left) and **2** (right). H atoms are omitted for clarity. Color code: Mn^II^, cyan; Mn^III^, blue; N, green; O, red; C, black.

**Figure 2:**
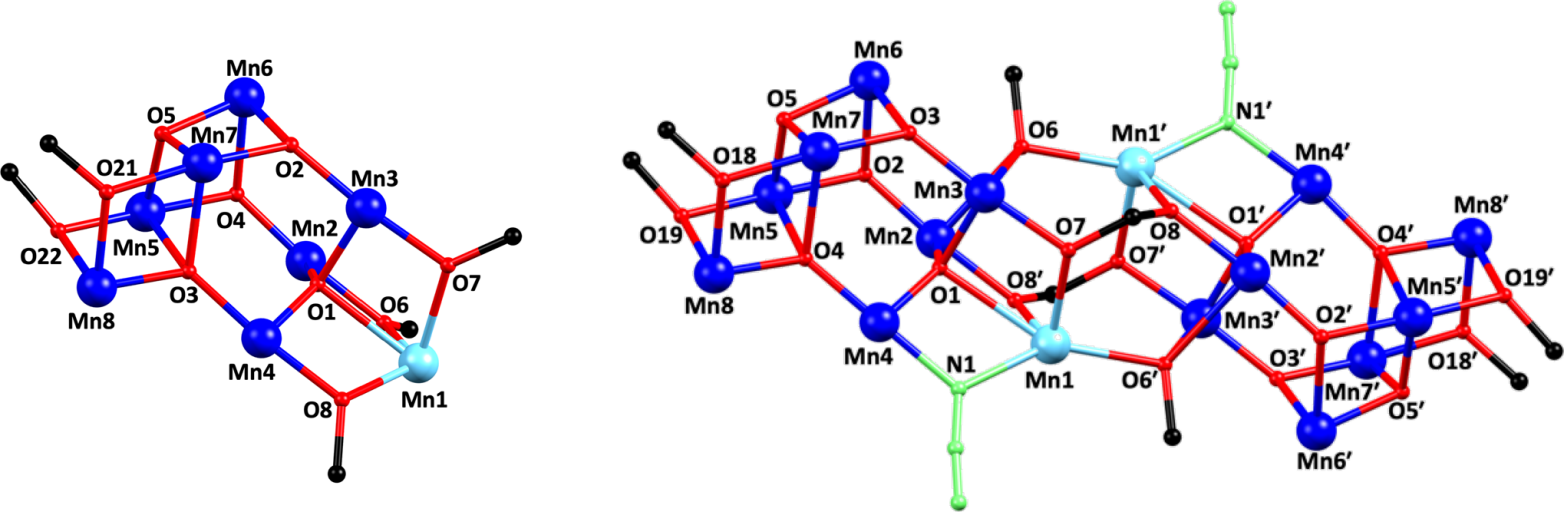
Partially labeled ball and stick representations of structural core of **1** (left) and **2** (right). H atoms are omitted for clarity. Color code: Mn^II^, cyan; Mn^III^, blue; N, green; O, red; C, black. Symmetry code: (′) -x, -y, -z.

**Scheme 2:**
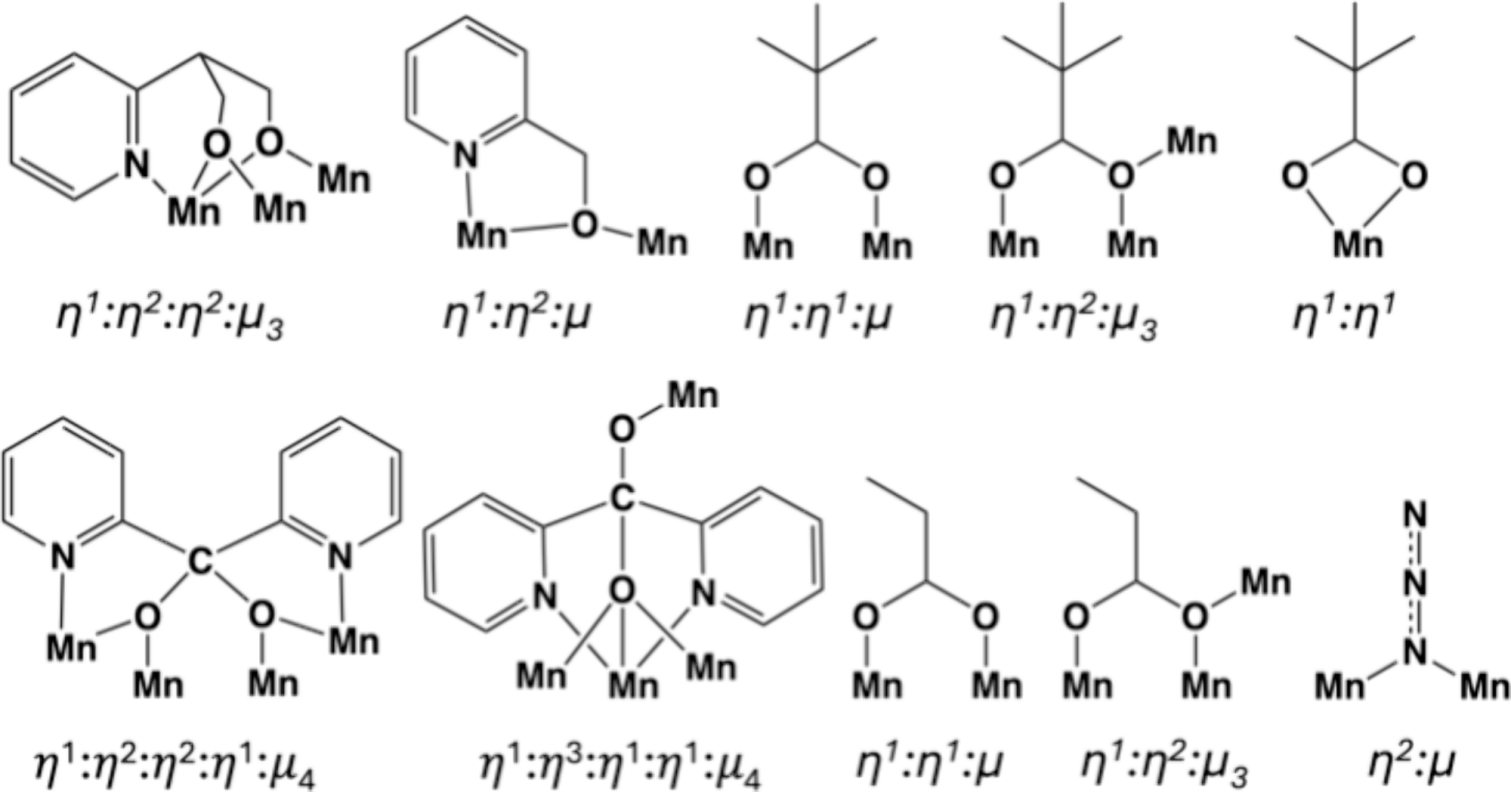
Crystallographically Established Coordination Modes of Ligands pypd^2–^, hmp^–^, (py)_2_CO_2_^2–^, Me_3_CCO_2_^–^, EtCO_2_^–^, and N_3_^–^ in Complexes **1** and **2**

Representations of the molecular structures and the structural cores of compounds **1** and **2** are shown in Figures 1 and 2, respectively. The coordination modes of the pypd^2–^, hmp^–^, (py)_2_CO_2_^2–^ and the carboxylate ligands are illustrated in Scheme 2. For both complexes the oxidation states of the Mn ions, and the protonation level of O atoms, were determined by charge-balance considerations, inspection of the metric parameters, BVS calculations^67,68^ (Table S2), and the observation of Jahn–Teller distortions for the octahedral Mn^III^ ions, which take the form of axial elongation (Figure S1).

**Figure 3:**
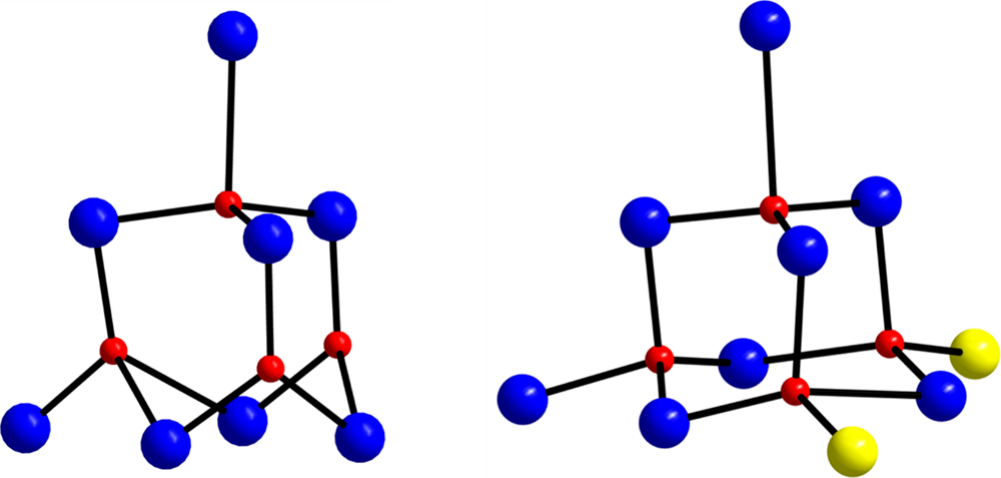
Defective supertetrahedral T3 Mn/O^2–^ core of **1** (left) and the typical decametallic supertetrahedral T3 core (right). Yellow color in the right representation is used to emphasize the missing metal ions from the defective supertetrahedral T3 Mn/O^2–^ core of **1**. Color code: Mn, blue; O, red.

Complex [Mn_8_Ο_5_(pypd)(hmp)_3_(O_2_CCMe_3_)_8_] ·0.5MeCN·0.5DMF·H_2_O (**1**·0.5MeCN·0.5DMF·H_2_O) crystallizes in the monoclinic space group *Cc* and its molecular structure consists of neutral cluster molecules containing the [Mn^III^_7_Mn^II^(μ_4_-O)_2_(μ_3_-O)_3_(μ-OR)_5_]^8+^ core. The latter consists of a bottom [Mn^III^_4_(μ_4_-O)(μ_3_-O)_3_(μ-OR)_2_]^2+^ rectangle connected to a [Mn^III^_3_Mn^II^(μ_4_-O)(μ-OR)_3_]^6+^ tetrahedral unit through one μ_4_- and two μ_3_-O^2–^ anions. The Mn^III^ ions of the [Mn^III^_4_(μ_4_-O)(μ_3_-O)_3_(μ-OR)_2_]^2+^ subunit lie in the same plane and are arranged in a defective dicubane conformation. The tetrahedral [Mn^III^_3_Mn^II^(μ_4_-O)(μ-OR)_3_]^6+^ subunit comprises one Mn^II^ and three Mn^III^ ions bridged by a central oxido μ_4_-O^2–^ ion and three μ-OR^–^ groups of hmp^–^ ligands. Alternatively, the core of **1** can be described as a supertetrahedron T3 that is missing two apexes (Figure 3). The 5 μ-OR^–^ bridges are provided by three η^1^:η^2^:μ hmp^–^ groups and one doubly deprotonated η^1^:η^2^:η^2^:μ_3_ pypd^2–^ ligand. Additional ligation is provided by eight pivalate ligands five of which bridge in the common *syn, syn-*η^1^:η^1^:μ fashion, two with a η^1^:η^2^:μ_3_ fashion and one acting as a chelating ligand. The unit cell of **1** also contains half MeCN, half DMF and one H_2_O solvent molecules. All Mn^III^ ions are six-coordinate, displaying a distorted octahedral coordination geometry. The geometry of the seven-coordinate Mn^II^ ion (Mn1) was estimated to be a distorted capped trigonal prismatic (Figure S2 and Table S3) using the SHAPE program (CShM value = 3.51).^69^ There are no direct intermolecular hydrogen bonding interactions between neighboring [Mn_8_] clusters, while the closest intermolecular metal···metal separation is 8.255 Å appearing between Mn2 and Mn7 centers.

Complex [Mn_16_Ο_10_(N_3_)_2_(pypd)_2_{(py)_2_CO_2_}_4_(O_2_CEt)_12_] ·MeCN (**2**·MeCN) crystallizes in the monoclinic space group *P*2_1_*/n*. Its molecular structure features a neutral centrosymmetric cluster containing a [Mn^III^_14_Mn^II^_2_(μ_4_-O)_4_(μ_3_-O)_6_(μ-N_3_)_2_(μ_3_-OR)_2_(μ-OR)_8_]^14+^ core (Figure 2). The core comprises two connected, symmetry-related [Mn^III^_7_Mn^II^(μ_4_-O)_2_(μ_3_-O)_3_(μ-N_3_)(μ-OR)_5_]^7+^ subunits that are similar to the structural core of complex **1**. The main differences between the subunit of complex **2** and the core of complex **1** include: (i) a μ-N_3_^–^ group in the place of one μ-OR bridge in the tetrahedral part of the subunit, bridging Mn1 and Mn4 centers; (ii) an additional μ-OR (O6) bridge in the tetrahedral part of the subunit of **2**, connecting Mn2 and Mn3 centers; and (iii) a change of the coordination geometry of the seven-coordinate Mn1 ion which is intermediate between capped octahedral (CShM value = 2.48) and capped trigonal prismatic (CShM value = 2.56) in **2** as indicated in Table S3 and Figure S2. The two [Mn_8_] subunits in complex **2** are connected via the alkoxido arms of four (py)_2_CO_2_^2–^ ligands. In particular, the apex Mn^II^ ion (Mn1) of one subunit is connected through a μ_3_-OR^–^ group (O6) to two Mn^III^ ions (Mn2′ and Mn3′) of the [Mn^II^Mn^III^_3_(μ_4_-O)(μ-N_3_)(μ-OR)_3_]^5+^ tetrahedron of the other subunit. The remaining two (py)_2_CO_2_^2–^ ligands link Mn1 and Mn3 of one subunit with Mn1′ and Mn2′ of the other subunit via their μ-OR^–^ groups (O7 and O8). The complete core features eight μ-OR and two μ_3_-OR bridges, provided by two η^1^:η^3^:η^1^:η^1^:μ_4_ and two η^1^:η^2^:η^2^:η^1^:μ_4_ (py)_2_CO_2_^2–^ ligands, along with two η^1^:η^2^:η^2^:μ_3_ pypd^2–^ groups. Additional ligation is provided by 12 bridging propionate ligands, ten of which adopt the common *syn,syn-*η^1^:η^1^:μ coordination mode and the remaining two the η^1^:η^2^:μ_3_ one. The unit cell of **2** also contains one MeCN solvent molecule. All Mn ions are six-coordinate with distorted octahedral geometries, except for Mn1, which is seven-coordinate. An examination of the packing of compound **2** revealed that there are no direct intermolecular hydrogen bonding interactions between neighboring [Mn_16_] complexes. The closest intermolecular metal···metal separation is 7.234 Å between Mn8 centers of adjacent [Mn_16_] clusters.

To the best of our knowledge, complexes **1** and **2** are the first structurally characterized Mn complexes containing the neutral or anionic forms of pypdH_2_.

### 3.3 DC Magnetic Measurements

**Figure 4:**
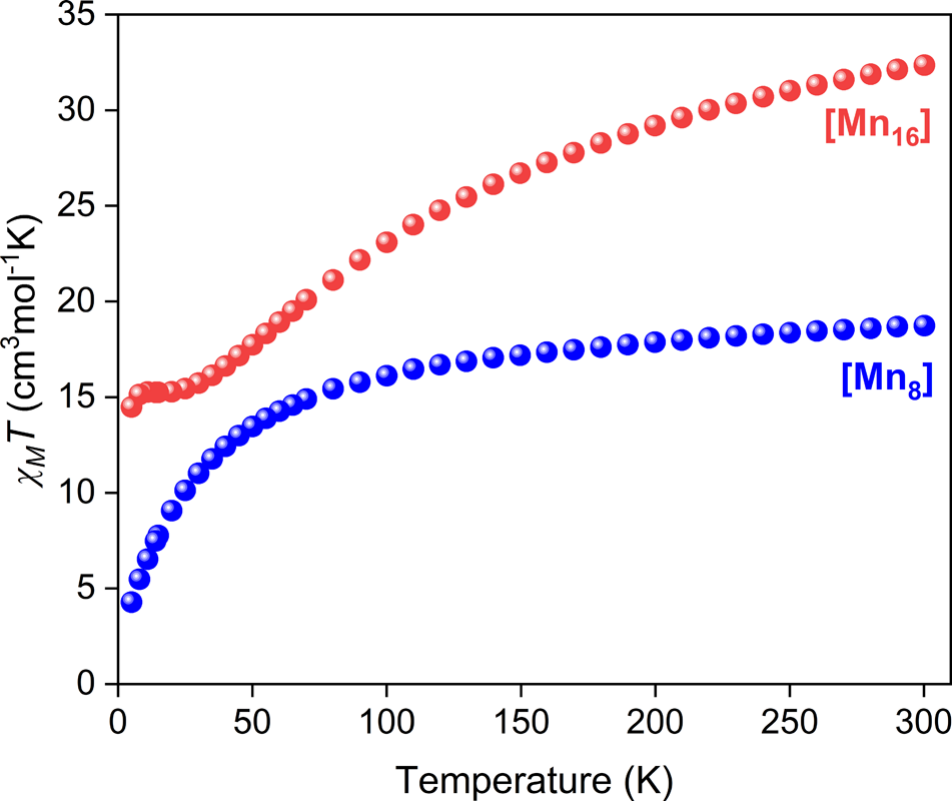
Temperature dependence of χ_M_*T* product for complexes **1** and **2** in a field of 0.1 T.

Solid-state, variable-temperature direct current (dc) magnetic susceptibility measurements were performed on powdered polycrystalline samples of **1** and **2** in a 0.1 T field and in the 5.0–300 K range. The χ_M_*T* versus *T* plots for the two complexes are depicted in Figure 4.

For complex **1**, the χ_M_*T* product steadily decreases from 18.73 cm^3^ mol^–1^ K at 300 K to 13.46 cm^3^ mol^–1^ K at ∼50 K, and then it rapidly drops to a minimum of 4.28 cm^3^ mol^–1^ K at ∼5.0 K. The χ_M_*T* at 300 K is smaller than the spin-only (*g* = 2.0) value of 25.38 cm^3^ mol^–1^ K for one Mn^II^ and seven Mn^III^ noninteracting ions, indicating the presence of dominant antiferromagnetic exchange interactions even at room temperature. The χ_M_*T* at ∼5.0 K suggests a small spin ground state value (either 3/2 or 5/2).

For complex **2**, the χ_M_*T* steadily decreases from 32.36 cm^3^ mol^–1^ K at 300 K to 16.62 cm^3^ mol^–1^ K at ∼40 K, and then it plateaus until ∼15 K before it drops to a minimum value of 14.48 cm^3^ mol^–1^ K at ∼5.0 K. The χ_M_*T* at 300 K is much smaller than the spin-only (*g* = 2.0) value of 50.75 cm^3^ mol^–1^ K for two Mn^II^ and 14 Mn^III^ noninteracting ions. This, as well as the shape of the χ_M_*T* curve indicate the presence of predominant antiferromagnetic exchange interactions. The χ_M_*T* at ∼5.0 K suggests a spin ground state value of *S*_T_ ∼ 5.

### 3.4 AC Magnetic Measurements

To obtain additional information about the spin ground state, *S*_T_, values and the potential presence of slow relaxation of magnetization (indicative of SMM behavior) in complexes **1** and **2**, alternating current (ac) magnetic susceptibility studies were performed. Data were collected using a 3.5 G ac field and 50, 250, and 1000 Hz oscillation frequencies over the temperature range 1.8–15 K.

**Figure 5:**
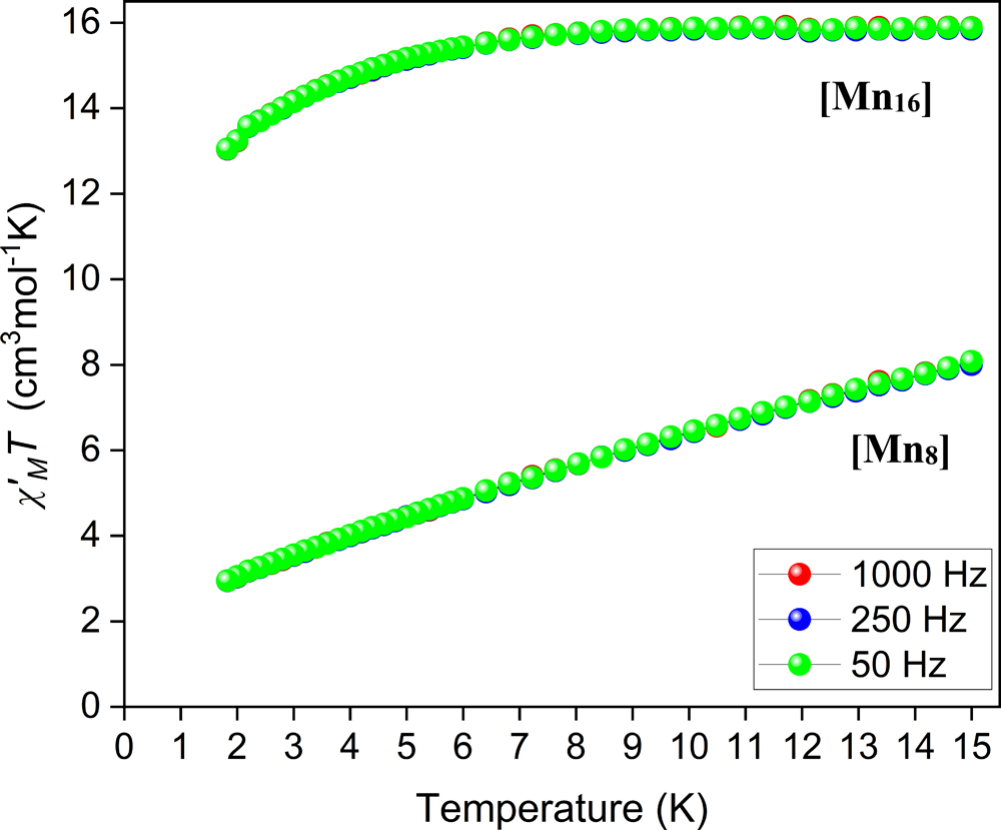
Plots of the in-phase (χ*′*_M_) signal as χ*′*_M_*T* vs *T* in a 3.5 G ac field oscillating at the indicated frequencies for complexes **1** and **2**.

For complex **1**, the in-phase signal (plotted as χ*′*_M_*T* vs *T*, Figure 5) decreases linearly from ∼8 cm^3^ mol^–1^ K at 15 K to ∼3 cm^3^ mol^–1^ K at ∼3 K. The observed decrease of the χ*′*_M_*T* with decreasing temperature is due to depopulation of excited states with spin S larger than that of the ground state. Extrapolation of χ*′*_M_*T* to 0 K using the data above 7 K gives a value of ∼3.5 cm^3^ mol^–1^ K, indicative of a spin ground state of *S*_T_ 3/2 or 5/2, in agreement with the conclusions derived from the dc studies. Owing to the small *S*_T_ value, complex **1** does not show any frequency-dependent out-of-phase ac signals down to 1.8 K (Figure S8, left), indicating that this compound does not exhibit SMM behavior.

For complex **2**, the in-phase signal (plotted as χ*′*_M_*T* vs *T*, Figure 5) is essentially constant with decreasing temperature from 15.89 cm^3^ mol^–1^ K at 15 K to 15.69 cm^3^ mol^–1^ K at 7 K. Below 7 K, the χ*′*_M_*T* product decreases more steeply reaching the value of 13.04 cm^3^ K mol^–1^ K at 1.8 K. Extrapolation of χ′_M_*T* to 0 K using the data above 7 K gives a value of 15.7 cm^3^ mol^–1^ K suggesting an *S*_T_ ∼ 5 ground state value with *g* ∼ 2.0. These findings are consistent with the results obtained from the dc data. Additionally, no out-of-phase χ″_M_ signals were observed for complex **2** (Figure S8, right), indicating that this compound does not exhibit SMM behavior.

Despite the presence of a nonzero spin ground state in complexes **1** and **2**, neither exhibits SMM behavior likely due to the absence of significant magnetic anisotropy. One possible reason for this could be the small value of the zero-field splitting (ZFS) parameter (D) because of the nonparallel alignment of the Jahn–Teller elongation axes of the high-spin octahedral Mn^III^ ions in these complexes (Figure S1).

## 4 Conclusions

The combined use of pypdH_2_ with either hmpH or (py)_2_CO ligands in manganese carboxylate chemistry has led to the successful synthesis of the two new mixed-valence polynuclear Mn clusters [Mn_8_Ο_5_(pypd)(hmp)_3_(O_2_CCMe_3_)_8_] (**1**) and [Mn_16_Ο_10_(N_3_)_2_(pypd)_2_{(py)_2_CO_2_}_4_(O_2_CEt)_12_] (**2**). Complexes **1** and **2** feature related and unique structural core topologies. Complex **1** exhibits a defective supertetrahedral T3 core missing two apexes, while complex **2** consists of two [Mn_8_] subunits that are similar to complex **1** thus, being the dimeric analogue of **1**. Direct current (dc) magnetic susceptibility studies revealed the presence of dominant antiferromagnetic interactions between the Mn ions in both complexes, resulting in small, ground spin state values of *S*_T_ 3/2 or 5/2 and 5 for **1** and **2**, respectively. These findings highlight the potential of polyol-type ligands to stabilize high nuclearity clusters based on polynuclear building blocks; in this case not only the oligomeric compound but also the building block display unprecedented metal/O core topologies. In addition, this work provides an initial proof of the capability of pypdH_2_ ligand to produce Mn clusters with novel structural motifs. Further studies involve a more detailed investigation of the use of pypdH_2_ ligand in metal cluster chemistry, either alone or in combination with other hydroxyl-containing ligands.

## Supporting Information Available

Crystal data and refinement parameters; BVS calculations; structural figures; SHAPE measurements, pXRD patterns, spectroscopic (IR), TGA, and magnetism plots for complexes **1** and **2;** and crystal structures of complexes **1** and **2** (PDF)
