# Supplementary material for: A [Mn8] Defective Supertetrahedron T3 and Its Dimeric [Mn16] Analogue
Source: Cryst Growth Des. 2024 Oct 9;24(21):9076–83. doi: 10.1021/acs.cgd.4c01100 (PMC11555654; doi:10.1021/acs.cgd.4c01100)
Supplement: Supplementary file 1 — cg4c01100_si_001.pdf [file cg4c01100_si_001.pdf]

SUPPORTING INFORMATION (SI)

A [Mn_8] Defective Supertetrahedron T3 and its Dimeric [Mn_{16}] Analogue

Antonis Anastassiades,[a] Dimitris I. Alexandropoulos,[a,c] Ashlyn Hale,[b] George Christou,[b] Spyros P. Perlepes,[c] and Anastasios J. Tasiopoulos[a]*

[a] Department of Chemistry, University of Cyprus, Nicosia 1678, Cyprus

Email: atasio@ucy.ac.cy

[b] Department of Chemistry, University of Florida, Gainesville, Florida 32611, USA

[c] Department of Chemistry, University of Patras, 26504 Patras, Greece

Table of contents

Single crystal X-Ray crystallography 3

Physical Measurements / Characterization..7

Magnetic measurements 11

Single crystal X-Ray crystallography

Table S1. Crystal data and structural refinement parameters for compounds **1**·0.5MeCN·0.5DMF·H_2O and **2**·MeCN.

Complex	**1**·0.5MeCN·0.5DMF·H_2O	**2**·MeCN
Empirical formula	$C_{68.5}H_{106}Mn_8N_5O_{27.5}$	$C_{98}H_{113}Mn_{16}N_{17}O_{46}$
Formula weight	1879.10	3144.09
Temperature/K	180 (1)	158 (1)
Crystal system	monoclinic	monoclinic
Space group	Cc	P 2_1/n
a/Å	17.9393(2)	13.1518(3)
b/Å	27.5173(2)	19.6573(3)
c/Å	18.2865(2)	26.0463(4)
α/°	90	90
β/°	90.568(2)	100.328(2)
γ/°	90	90
Volume/ $Å^3$	9026.5(2)	6624.6(2)
Z	2	2
ρ_{calc} g/cm^3	1.383	1.576
μ/mm^{-1}	9.406	12.662
F(000)	3888.0	3176.0
Crystal size/mm^3 (max x mid x min)	0.247 x 0.18 x 0.032	0.307 x 0.13 x 0.021
Radiation / Å	Cu Kα λ=1.54184	Cu Kα λ=1.54184
2Θ range for data collection / °	5.882 to 134.134	6.9 to 154.998
Index ranges	-21 ≤ h ≤ 21<br>-32 ≤ k ≤ 32<br>-20 ≤ l ≤ 21	-16 ≤ h ≤ 16<br>-24 ≤ k ≤ 24<br>-32 ≤ l ≤ 32
Reflections collected	50477	51075
Independent reflections	11221 [R(int) = 0.0504]	13563 [R(int) = 0.0724]
Data/restraints/parameters	11221/236/1049	13563/100 /847
Goodness-of-fit on F^2	1.044	1.025
Final R indices [I>2σ(I)]	R_1^a = 0.0574<br>wR_2^b = 0.1577	R_1^a = 0.0818<br>wR_2^b = 0.2181
Final R indices [all data]	R_1 = 0.0599<br>wR_2 = 0.1603	R_1 = 0.1182<br>wR_2 = 0.2471
Largest diff. peak/hole /e $Å^{-3}$	1.15 and -1.22	1.46 and -1.07

$^aR_1 = \Sigma(||F_o| - |F_c||)/\Sigma|F_o|$. $^bwR_2 = [\Sigma[w(F_o^2 - F_c^2)^2]/\Sigma[w(F_o^2)^2]]^{1/2}$, $w = 1/[\sigma^2(F_o^2) + [(ap)^2 + bp]$, where $p = [\max(F_o^2, 0) + 2F_c^2]/3$.

Table S2. Bond valence sum (BVS)[a] calculations for Mn ions in **1** and **2**.

Complex **1**			
Atom	Mn(II)	Mn(III)	Mn(IV)
Mn1	<u>1.96</u>	1.81	1.88
Mn2	3.30	<u>3.05</u>	3.15
Mn3	3.31	<u>3.06</u>	3.16
Mn4	3.09	<u>2.82</u>	2.97
Mn5	3.13	<u>2.87</u>	3.01
Mn6	3.07	<u>2.81</u>	2.95
Mn7	3.10	<u>2.83</u>	2.97
Mn8	3.19	<u>2.95</u>	3.05
Complex **2**			
Atom	Mn(II)	Mn(III)	Mn(IV)
Mn1	<u>1.91</u>	1.80	1.81
Mn2	3.25	<u>3.01</u>	3.10
Mn3	3.31	<u>3.07</u>	3.16
Mn4	3.17	<u>2.94</u>	3.02
Mn4A	3.15	<u>2.92</u>	3.01
Mn5	3.12	<u>2.85</u>	2.99
Mn6	3.24	<u>2.96</u>	3.11
Mn7	3.17	<u>2.90</u>	3.04
Mn8	3.20	<u>2.96</u>	3.05

[a]The underlined value is the one closest to the charge for which it was calculated. The oxidation state is the nearest integer number to the underlined value.

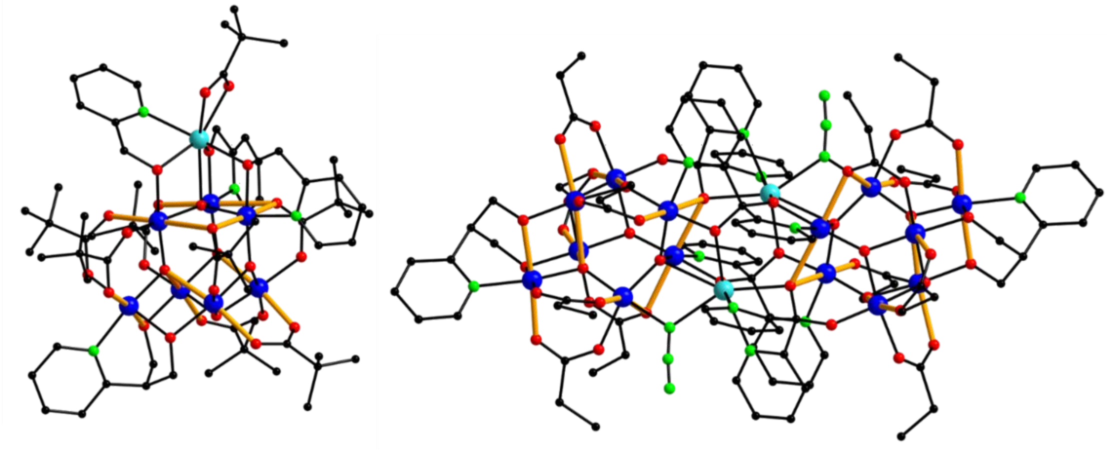

Fig. S1. Representations of the molecular structures of complexes **1** (left) and **2** (right) emphasizing the Jahn–Teller axes of Mn^{III} ions (bold orange solid lines). Color code: Mn^{II}, cyan; Mn^{III}, blue; N, green; O, red; C, black.

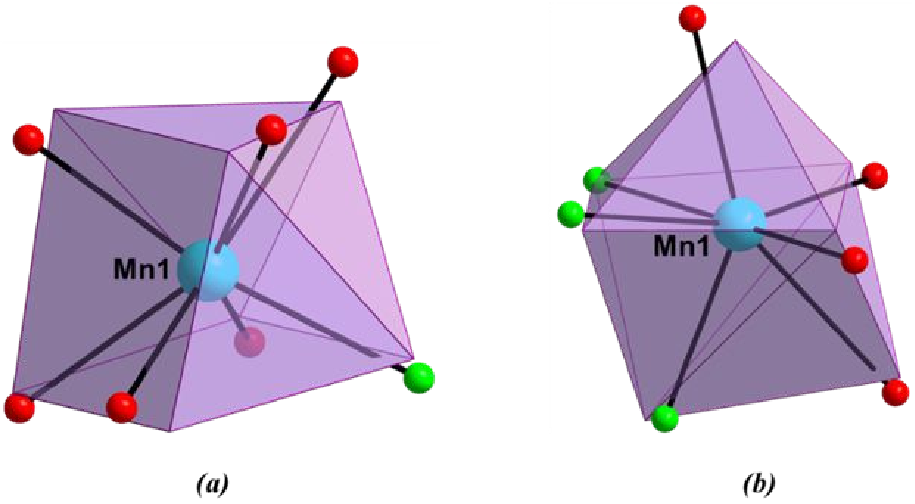

Fig. S2. Representations of the (a) capped trigonal prismatic coordination polyhedron of Mn1 in **1** and (b) capped octahedral coordination polyhedron of Mn1/1′ in **2**. The thin purple lines define the vertices of the ideal polyhedra. Colour code: Mn^{II}, cyan; N, green; O, red.

Table S3. Continuous Shape Measures (CShM) values for the potential coordination polyhedra of the 7-coordinate Mn1 centers in complexes **1** and **2**.

Polyhedron[a,b]	**1**	**2**
HP	30.60	31.73
HPY	17.62	18.47
PBPY	4.65	5.46
COC	3.92	**2.48**
CTPR	**3.51**	2.56
JPBPY	7.99	8.00
JETPY	14.58	15.28

[a]Abbreviations: HP, Heptagon; HPY, Hexagonal pyramid; PBPY, Pentagonal bipyramid; COC, Capped octahedron; CTPR, Capped trigonal prism; JPBPY, Johnson pentagonal bipyramid J13; JETPY, Johnson elongated triangular pyramid J7. [b]The values in boldface indicate the closest polyhedron according to Continuous Shape Measures.

Physical Measurements / Characterization

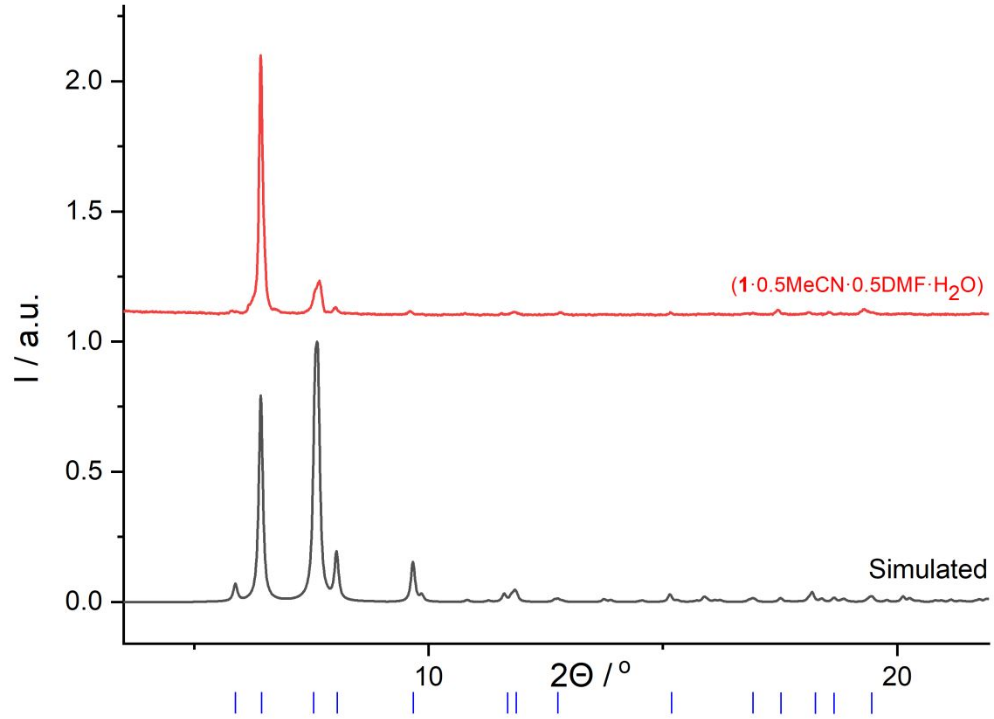

Fig. S3. Powder X-ray diffraction patterns of compound **1**, along with the simulated pattern from the single crystal data.

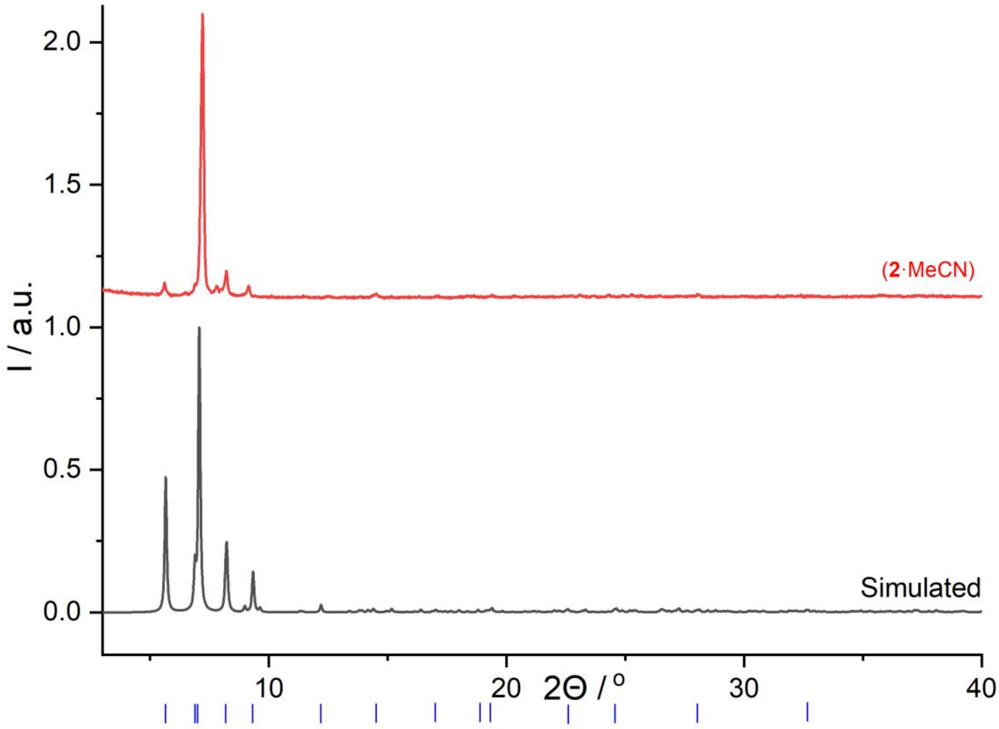

Fig. S4. Powder X-ray diffraction patterns of compound **2**, along with the simulated pattern from the single crystal data.

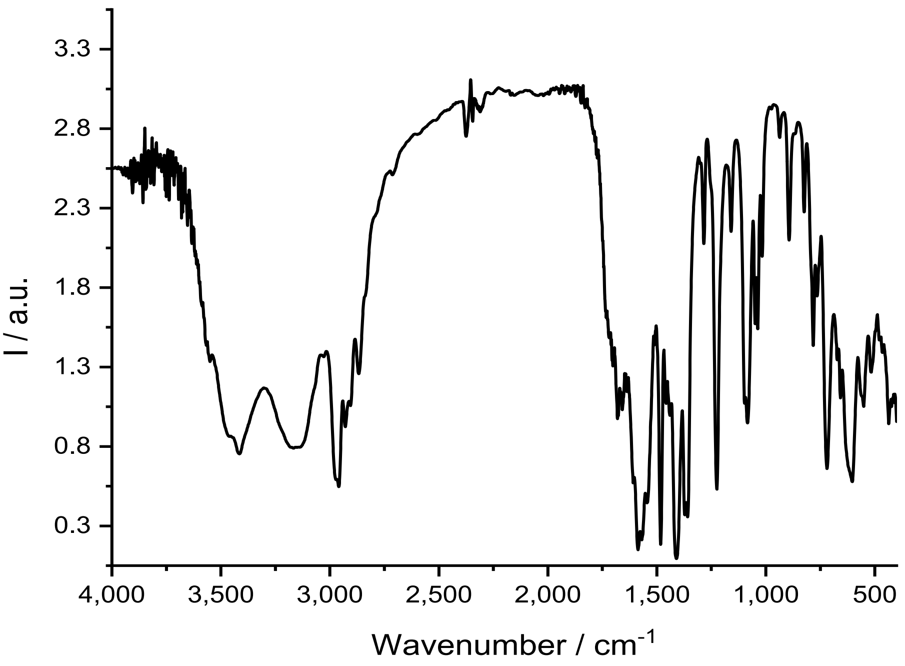

Fig. S5. FT-IR spectrum of complex **1**.

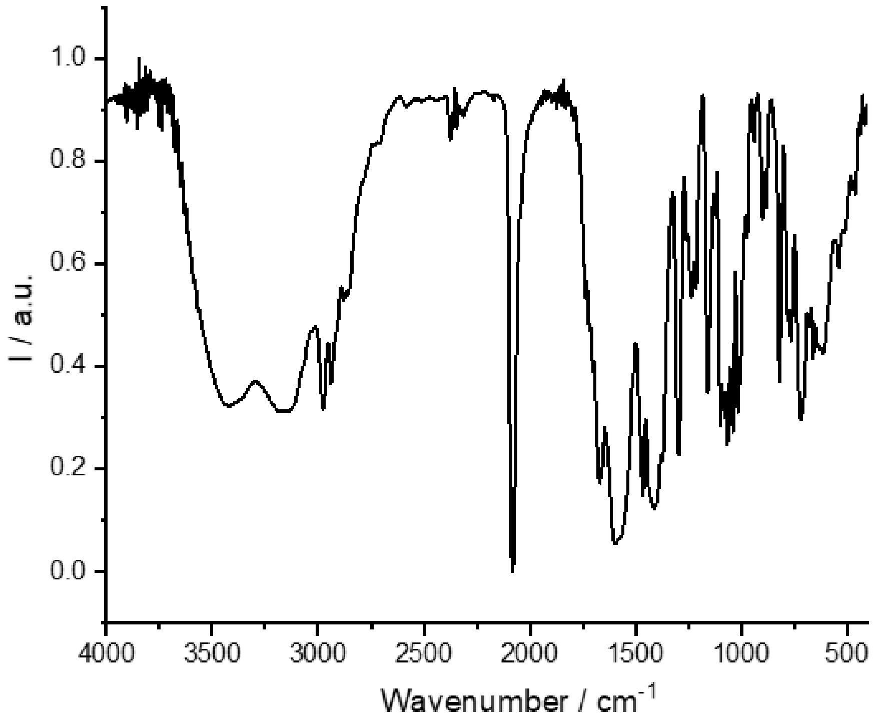

Fig. S6. FT-IR spectrum of complex **2**.

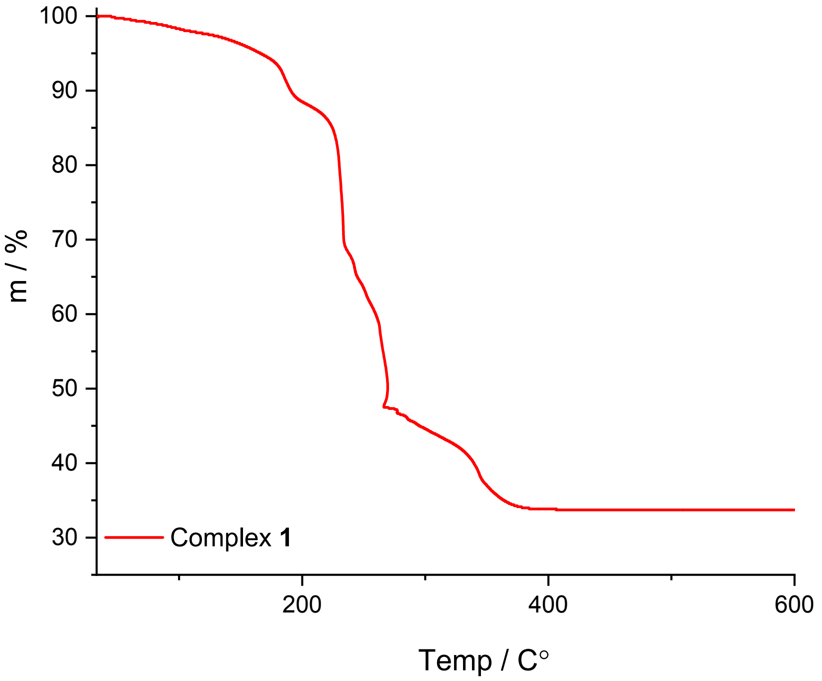

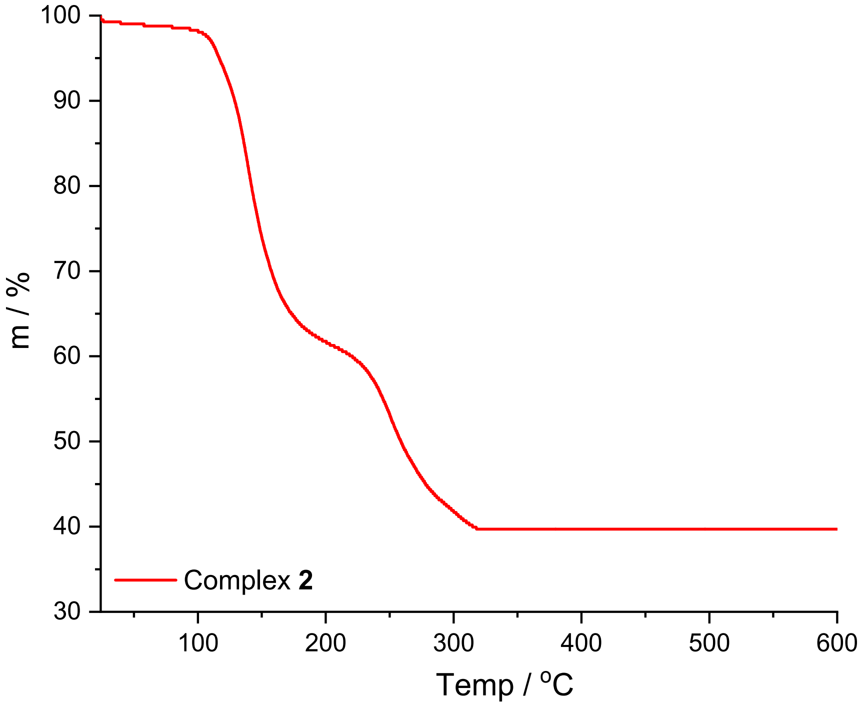

Fig. S7. TGA graphs of: (top) $[Mn_8O_5(pypd)(hmp)_3(O_2CCMe_3)_8]$ **(1)** and (bottom) $[Mn_{16}O_{10}(N_3)_2(pypd)_2\{(py)_2CO_2\}_4(O_2CEt)_{12}]$ **(2)**

Table S4. Calculated values for percentage mass loss of solvent removal and ligand combustion along with the experimental values obtained from TGA analysis of compound $[Mn_8O_5(pypd)(hmp)_3(O_2CCMe_3)_8]$ **(1)** and $[Mn_{16}O_{10}(N_3)_2(pypd)_2\{(py)_2CO_2\}_4(O_2CEt)_{12}]$ **(2)**:

Removal of Lattice Solvents				Ligand Combustion		Residual Oxide(s)		
Compound	Temperature (°C)	Experimental (Calculated) (%)	xMeCN yDMF zH_2O	Temperature (°C)	Experimental (Calculated) (%)	Temperature (°C)	Experimental (Calculated) (%)	Formula
**(1)**	r.t.-160	3.8 (4)	x=0.5 y=0.5 z=1	160-600	62.5 (62.8)	600	33.7 (33.2)	$MnO/3.5Mn_2O_3$
**(2)**	r.t.-80	1.5 (1.3)	x=1	80-600	58.8 (9)	600	39.7 (39.7)	$2MnO/7Mn_2O_3$

Magnetic measurements

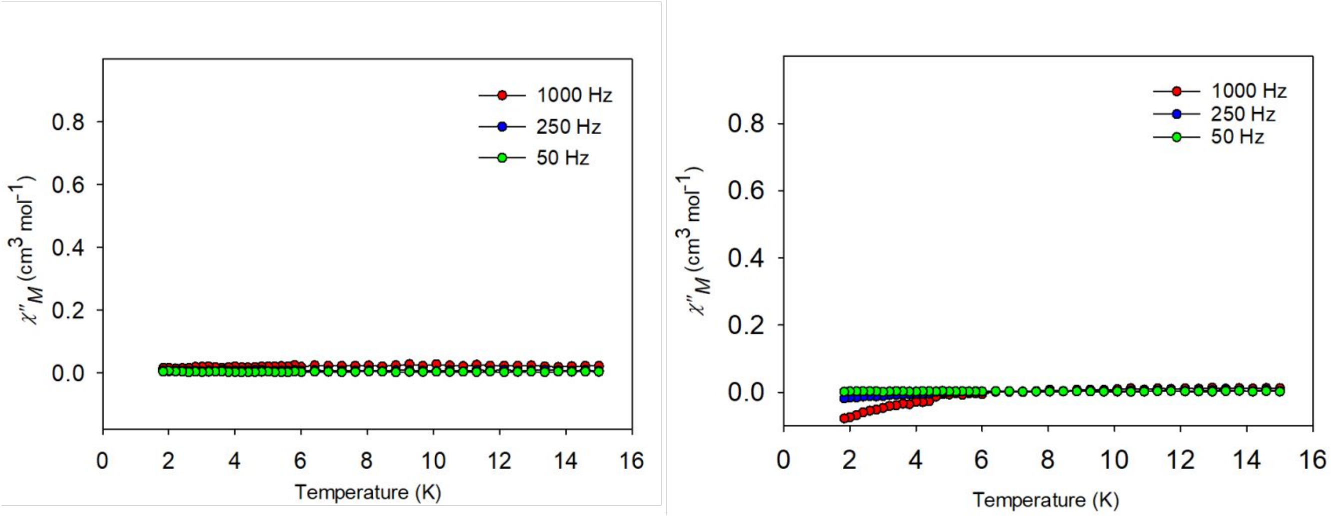

Figure S8. Temperature dependence of the out-of-phase χ''_M ac susceptibility signal of **1** (left) and **2** (right) in a 3.5 G field oscillating at the indicated frequencies.
